# A genome-wide imaging-based screening to identify genes involved in synphilin-1 inclusion formation in *Saccharomyces cerevisiae*

**DOI:** 10.1038/srep30134

**Published:** 2016-07-21

**Authors:** Lei Zhao, Qian Yang, Ju Zheng, Xuefeng Zhu, Xinxin Hao, Jia Song, Tom Lebacq, Vanessa Franssens, Joris Winderickx, Thomas Nystrom, Beidong Liu

**Affiliations:** 1 School of Life Science and Technology, Harbin Institute of Technology, Harbin 150001, China; 2 Department of Biology, Functional Biology, KU Leuven, 3001 Heverlee, Belgium; 3 Department of Medical Biochemistry and Cell Biology, University of Gothenburg, S-405 30, Göteborg, Sweden; 4 Department of Chemistry and Molecular Biology, University of Gothenburg, S-413 90, Göteborg, Sweden

## Abstract

Synphilin-1 is a major component of Parkinson’s disease (PD) inclusion bodies implicated in PD pathogenesis. However, the machinery controlling synphilin-1 inclusion formation remains unclear. Here, we investigated synphilin-1 inclusion formation using a systematic genome-wide, high-content imaging based screening approach (HCI) in the yeast *Saccharomyces cerevisiae*. By combining with a secondary screening for mutants showing significant changes on fluorescence signal intensity, we filtered out hits that significantly decreased the expression level of synphilin-1. We found 133 yeast genes that didn’t affect synphilin-1 expression but that were required for the formation of synphilin-1 inclusions. Functional enrichment and physical interaction network analysis revealed these genes to encode for functions involved in cytoskeleton organization, histone modification, sister chromatid segregation, glycolipid biosynthetic process, DNA repair and replication. All hits were confirmed by conventional microscopy. Complementation assays were performed with a selected group of mutants, results indicated that the observed phenotypic changes in synphilin-1 inclusion formation were directly caused by the loss of corresponding genes of the deletion mutants. Further growth assays of these mutants showed a significant synthetic sick effect upon synphilin-1 expression, which supports the hypothesis that matured inclusions represent an end stage of several events meant to protect cells against the synphilin-1 cytotoxicity.

Increasing evidence shows that neurodegenerative diseases such as Parkinson’s disease (PD), Alzheimer’s disease (AD), and Huntington’s disease (HD) share common underlying molecular and cellular mechanisms and that protein inclusion body formation is a typical hallmark for these diseases. Parkinson’s disease, which affects about 2% of the population over 65 years old, is an age-associated neurodegenerative disease that is characterized by degeneration of dopaminergic neurons and the formation of intracellular protein inclusions called Lewy bodies. It has been shown that the protein alpha-synuclein is the major component in these Lewy bodies^1^. In addition to alpha-synuclein, the presynaptic protein synphilin-1 was also found as one of the constituents of Lewy bodies^2^. Synphilin-1 not only interacts with alpha-synuclein^3^ but also with other proteins involved in the pathogenesis of PD, such as parkin and LRRK2^4^,^5^. This suggests that it may link several PD related gene products to a common pathogenic mechanism based on protein aggregation. Hence, a systematic analysis of mechanisms governing synphilin-1 inclusion formation will increase our understanding of the pathogenesis of PD.

Synphilin-1, encoded by the gene SNCAIP, contains 919 amino acids and 3 characterized domains: two protein-protein interaction domains (ankyrin-like repeats and coiled coil domain) and a predicted ATP/GTP-binding motif 6. However, the physiological functions of synphilin-1 are still not fully understood. When expressed in yeast synphilin-1 forms aggregates, similar as in mammalian systems^7^. To further investigate the mechanisms involved in synphilin-1 inclusion body formation, we performed a genome-wide imaging-based screening to isolate yeast deletion mutants that altered the formation of synphilin-1 inclusions. To this end, we applied the yeast synthetic genetic array (SGA) methodology^8^,^9^,^10^ and further developed a yeast high-content imaging based screening (HCI) approach to monitor the synphilin-1 inclusion phenotype. Several groups are currently applying yeast HCI screening approaches to investigate a wide range of biological questions. The pioneer proof-of-principle studies from Vizeacoumar and colleagues^11^ applied their high-content screening system to examine mitotic spindle morphogenesis. Recent results from the same group provided a proteome scale evaluation of protein abundance and localization in the cell^12^. Studies using the HCI approach have been performed in other research groups as well. Yang and colleagues investigated the machinery for the formation of stress granules as well as inclusion bodies (IB) by the misfolded Von Hippel–Lindau tumor suppressor protein (VHL)^13^,^14^. Combined, these results demonstrate that the yeast HCI approach is a powerful tool for investigating mechanisms underlying different cellular processes.

The results of our current study led us to identify the interaction networks and components involved in the formation of synphilin-1 inclusions. The synthetic sick effect, observed from the identified mutants by expressing synphilin-1, supports the hypothesis that synphilin-1 inclusion formation has a cytoprotective effect to the cell. These results provide a molecular basis for understanding potential roles of synphilin-1 in the pathogenesis of PD.

## Results and Discussion

**Figure 1:**
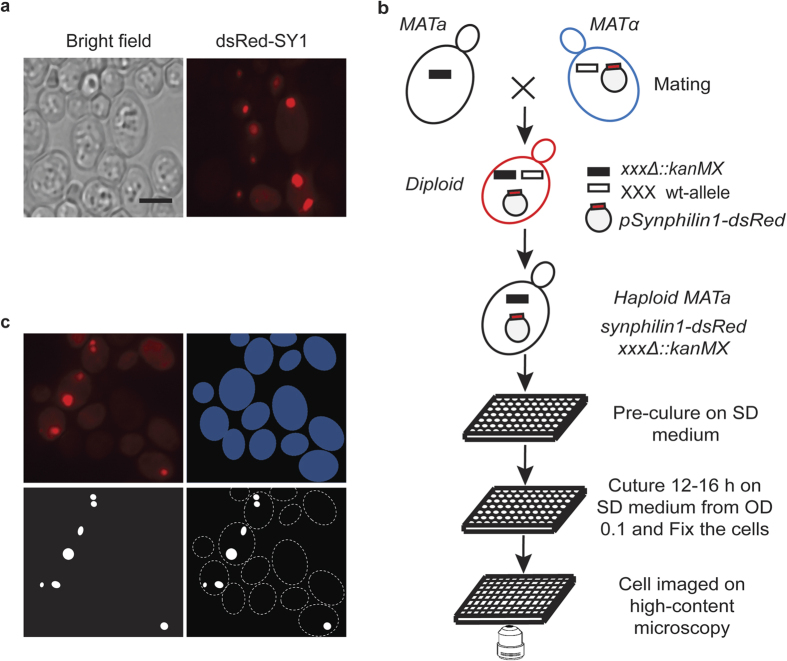
Genome-wide high-content imaging approach to identify components involved in synphilin-1 (SY1) inclusion formation. (**a**) Phenotype of WT cells harboring synphilin-1 inclusions. Right panel: dsRed-tagged synphilin-1; Left panel: Bright field. Scale bar, 5 μm. (**b**) Workflow of the high-content imaging based screening (HCI) to identify mutants altering the synphilin-1 inclusion formation. (**c**) Software approach for synphilin-1 inclusion identification and quantification. Image pre-processing with shade correction and background subtraction (Top left); Cells (Top right) and inclusion bodies (Bottom left) were isolate from their background based on the signal intensity differences. Then the percentage of cells with inclusions were extracted (Bottom right) by combining the two (cells and inclusion bodies) masks.

It was previously demonstrated that expression of the N-terminal fusion protein dsRed-synphilin-1 leads to inclusion (aggregate) formation in yeast cells, similarly to expression of WT-synphilin-1. This indicates that synphilin-1 is being processed in a similar way in yeast and mammalian cells^7^,^15^. To identify components regulating synphilin-1 inclusion formation *in cellulo*, we performed a yeast genome-wide high-throughput imaging screening. The plasmid pYX212 carrying dsRed-synphilin-1 under the control of constitutive *TPI1* promoter was introduced into yeast Y7092 query strains. The morphology of the synphilin-1 inclusions was tested manually as shown in Fig. 1a.

The setup of the robotic screening system is shown in Fig. 1b. This high content imaging screening system was designed to explore inclusion phenotypes through assessing fluorescent labeled protein markers in the yeast single deletion collection (SGA-V2) and the essential gene temperature sensitive (ts) allele collection (ts-V5) that contains yeast mutants harboring ts alleles of the essential genes. To incorporate the control plasmid expressing only dsRed or the plasmid for dsRed-synphilin-1 into the collections, we applied the yeast synthetic genetic array (SGA) methodology^8^,^9^,^10^. In this robotic procedure, standard mating and meiotic recombination is used to select the haploid cells that combine a specific deletion or ts-mutant allele with the presence of the desired plasmid (Fig. 1b). We also designed a scoring program to automatically process the large amount of acquired images. It has the ability to automatically query and identify the cells on the images, extract the number of cells carrying inclusion bodies and export the measurements as a separate output (Fig. 1c). The phenotype scored in this screening is the percentage of cells carrying inclusions formed by dsRed-synphilin-1.

There is a possibility that certain mutants would show an altered inclusion formation phenotype because of changes in the synphilin-1 expression level. To address this possibility, we also monitored steady-state levels of synphilin-1 by measuring the dsRed-synphilin-1 fluorescence intensity on the acquired images. Mutants with no significant difference (P > 0.05, Student’s *t*-test) in their fluorescence level compared to the wild-type cells are more likely to represent strains affected in the cellular machinery that supports inclusion formation and aggregation.

The criteria to include a particular mutant strain as a potential hit is based on the following: 1) the difference in the number of cells with synphilin-1 inclusions between wild type and mutant cells is statistically significant (P ≤ 0.05, Student’s *t*-test), 2) the absolute difference of the percentage of wild type and mutant cells carrying synphilin-1 inclusions must be at least 25% and 3) no significant (P > 0.05, Student’s *t*-test) fluorescence intensity changes can be observed between wild type and mutant cells. Potential hits acquired from the screening were confirmed for their aggregation phenotype by using a manual microscopy approach. Based on these criteria and our manual confirmation, a total of 148 confirmed hits were obtained from the screening of the SGA collection and the ts collection (totally 5155 yeast mutants, including 4368 single deletion mutants and 787 ts alleles). Of these, 15 mutants showed an increase of cells carrying synphilin-1 inclusions, but most of them also display a significantly increased synphilin-1 expression level as measured by fluorescence intensity. The remaining 133 mutants showed a decrease in the percentage of cells carrying synphilin-1 inclusions without significant changes in the signal intensity of synphilin-1 (Supplementary Tables S3 and S6). Among these, 100 were non-essential gene deletion mutants and 33 were essential gene ts alleles. For the mutants with a decrease in synphilin-1 inclusion formation, the confirmation rate obtained by this manual microscopic inspection was 92.9% when using a cutoff of 25% difference between the wild type and the mutants for the number of cells with synphilin-1 inclusions. This means that the automated image analysis selected about 7.1% false positives, but this number could be reduced further by selecting higher cutoff values (Supplementary Fig. S1).

Mutants showing significant decrease (P ≤ 0.05, Student’s *t*-test) in both the percentage of cells with synphilin-1 inclusions and the fluorescence intensity are listed in Supplementary Table S8. For these mutants, there is a possibility that the observed decreased inclusion formation phenotype is caused by the low expression level of the synphilin-1 protein.

### Mutants displaying a decreased capacity to form synphilin-1 inclusions

**Figure 2:**
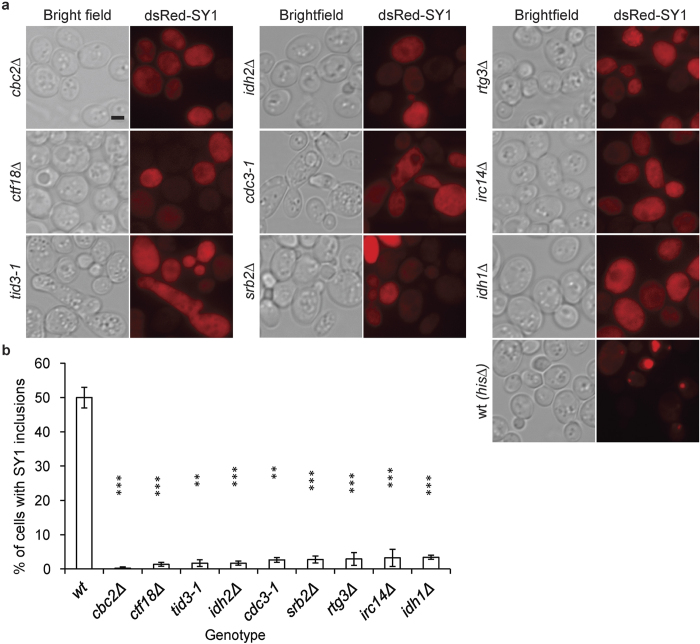
Top hits showing a decreased percentage of cells with synphilin-1 (SY1) inclusions obtained from the HCI screening. (**a**) Images of mutants displaying less synphilin-1 inclusions than WT (*his3Δ*) cells. Left panel: bright field, right panel: dsRed-synphilin-1. Scale bar, 2 μm. (**b**) Quantification of the percentage of cells with inclusions in WT and mutant strains. Values are normalized such that the percentage of cells with inclusions in WT is set to 50%. A value of 0% means that there are no cells of that mutant with synphilin-1 inclusions. Statistical analysis was performed using Student’s *t*-test. Error bars represent standard deviation from triplicates of about 200 cells each. Asterisks denote significant differences between WT and mutants: *P, 0.05; **P, 0.01; ***P, 0.001.

**Figure 3:**
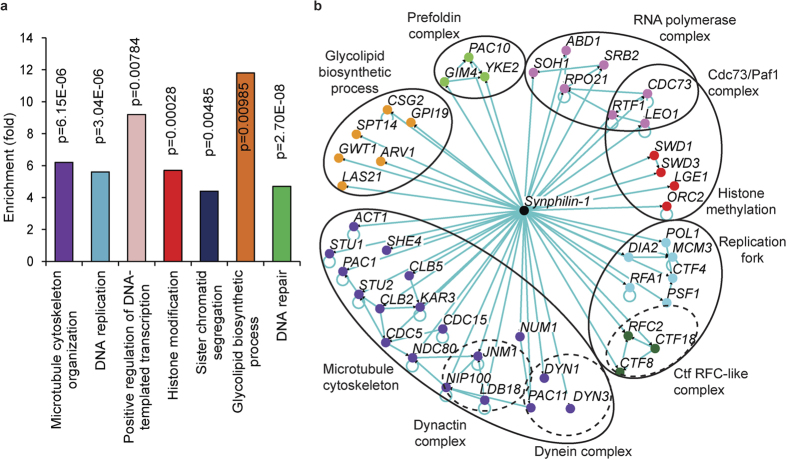
Functional enrichment and network analysis for the confirmed hits with decreased synphilin-1 inclusion formation. (**a**) Functional enrichment analysis of mutants with decreased synphilin-1 inclusion formation. Confirmed mutants were analyzed for enrichment of GO functional categories. Enriched groups were scored by comparing to a background list of SGA-V2+ ts-V5 array using a cut-off of P ≤ 0.05. Functional groups are marked with different colors. (**b**) Network analysis of mutants with decreased synphilin-1 inclusion formation. Mutants showing less cells with synphilin-1 inclusion were grouped into modules based on their known physical interactions and published information of the cellular components. The cellular components are shown in different colored nodes. The circles indicate subunits or protein complexes.

Representative images of some top hits with high significant differences on decreased inclusion formation by dsRed-synphilin-1 are shown in Fig. 2a, and their quantitative differences as compared to the wild type strain are presented in Fig. 2b. Next, the mutants were analyzed for the enrichment of Gene Ontology (GO) categories, which classified them into different functional groups, including cytoskeleton organization, histone modification, sister chromatid segregation, glycolipid biosynthetic process, DNA repair, and DNA replication (Fig. 3a, Supplementary Table S4). Within these functional groups several protein complexes were identified such as the dynein complex, dynactin, prefoldin complex, the Ctf18 RFC-like complex, the RNA polymerase complex and the Cdc73/Paf1 complex (Fig. 3b). Overall, this analysis revealed the complexity of the machinery involved in the formation of dsRed-synphilin-1 inclusions. Furthermore, we identified the human homologues of these enirched hits using YeastMine web-based software (a multifaceted search and retrieval system)^16^. The results are shown in Supplementary Table S5.

### Genes involved in cytoskeleton organization, dynein, dynactin and prefoldin complex

It has previously been reported that the actin cytoskeleton is involved in synphilin-1, Huntingtin and heat induced protein inclusion/aggregate management^7^,^17^,^18^,^19^. Consistently, the GO term analysis showed a moderate enrichment for gene products involved in cytoskeleton organization (12.0% vs 1.9%, relative vs background frequency, P = 4.9 × 10^−6^), which included genes such as *ACT1* (Fig. 3a,b). These genes are involved in cell polarization, endocytosis and other cytoskeleton-related functions. Also genes encoding for dynein motor components (*DYN1, DYN3,* and *PAC11*) and a microtubule plus-end binding protein (*PAC1*) were retrieved (Fig. 3a,b). Cytoplasmic dynein is responsible for transport of cargo along microtubules, organization of the microtubule network with respect to the cell cortex and positioning of microtubule organizing center^20^. There is also a considerable enrichment of genes encoding for the dynactin complex (2.4% vs 0.1%, relative to the background frequency, P = 0.013) including *LDB18*, *JNM1* and *NIP100* (Fig. 3a,b). Dynactin, a widely conserved multi-subunit complex, is necessary for the function of dynein in cytoplasm and takes part in a variety of microtubule-based transport and anchoring processes. It has also been found to interact with dynein, microtubules and various types of cargo^21^. Moreover, the movement and position of the mitotic spindle and nucleus is known to be impaired when dynactin is absent^22^. It has been suggested that inclusions tend to accumulate in inclusion bodies called aggresomes under proteasome inhibition and that the formation of aggresomes is based on dynein-dependent retrograde transport along microtubules^23^,^24^. In mammalian cells experimental inhibition of components of protein folding can induce aggregate formation^23^,^25^. In our screening, we found that prefoldin complex components (*GIM4, YKE2* and *PAC10*) as being required for proper synphilin-1 inclusion formation in yeast cells. This result not only confirms previously reported data^7^,^15^, but it is especially interesting because the prefoldin complex is a chaperone that delivers unfolded proteins to cytosolic chaperonin^26^, which has been implicated as a potent modulator of protein misfolding disease^27^. Further understanding of the role of prefoldin complex and its co-player chaperonin in synphilin-1 inclusion formation is likely to provide important insight into basic pathogenesis mechanisms of these proteins^27^. Cytoskeletal proteins are also believed to be involved in both formation and maintenance of yeast prions^28^,^29^,^30^. Whether the cytoskeletal components identified from our screening are involved in the formation of the inclusions or in their partitioning remains to be elucidated.

### Genes involved in sister chromatid cohesion

Our screening results also show that the formation of synphilin-1 inclusions is impinged in mutants involved in sister chromatid cohesion and spindle orientation function. GO term enrichment analysis showed that mitotic sister chromatid cohesion was about 5 fold enriched and this included genes encoding the Ctf18 RFC-like complex (*CTF18, CTF8, RFC2*) (Fig. 3a,b). The RFC complex is required for sister chromatid cohesion and chromosome transmission, and mutants carrying a *CTF8, CTF18*, or *DCC1* deletion display a severe sister chromatid cohesion defect^31^. It is possible that the establishment of sister chromatid cohesion plays a role in the formation of synphilin-1 inclusions. Moreover, the Ctf18 protein co-localizes with the replication fork during DNA replication where it has an important role for sister chromatid cohesion^32^. Related to this result, there was also a moderate enrichment for gene products involved in DNA replication initiation/Primase complex, such as *RFA1, PSF1, MCM3,* and *POL1* (Fig. 3a,b). However, for these mutants it is not clear whether the observed decrease in the inclusion formation phenotype of the corresponding mutants is functionally linked to the sister chromatid hits or whether it is simply due to a general reduction of DNA replication.

### Genes involved in glycolipid biosynthetic process

The list of genes whose deletion is associated with decreased synphilin-1 inclusion formation is also enriched in members of the glycolipid biosynthetic process (*CSG2, GPI19, LAS21, GWT1, ARV1 and SPT14*; 4.7% vs 0.4% in the background; P = 9.8 × 10^−3^; Fig. 3a,b). Five of them (*GPI19, LAS21, GWT1, ARV1 and SPT14*) are especially involved in the glycosylphosphatidylinositol (GPI) anchor biosynthetic process. Among these, *GPI19* and *SPT14* belong to the glycosylphosphatidylinositol-N- acetylglucosaminyltransferase (GPI-GnT) complex, which mediates the first step in GPI biosynthesis.

Defects in the GPI-anchor synthesis has been linked with congenital diseases such as hyperphosphatasia with mental retardation syndrome (HPMRS), also known as Mabry syndrome^33^. Moreover, GPI anchors have been identified as factors that are required for the neurotoxic effect of scrapie prions^34^. Here we show that the GPI anchor biosynthetic process components are required for synphilin-1 inclusion formation, whether this indicates that there is a direct interaction between synphilin-1 and GPI anchors needs to be tested. It is known that the synphilin-1 associates with cell membrane structures such as lipid droplets and lipid rafts in mammalian and yeast cells^7^,^35^. Further in-depth investigation on the interplay among synphilin-1, GPI-anchor and cell membrane structures will provide novel clues on the pathological role of synphilin-1.

### Genes involved in Cdc73/Paf1 complex

We also found several mutants carrying deletions of genes belonging to the Cdc73/Paf1 complex that regulates transcription elongation from RNA polymerase (*rtf1Δ*, *cdc73Δ*, *leo1Δ*; 2.4% vs 0.1% in the background, P = 3.5 × 10^−3^; Fig. 3a,b). This complex was initially identified as the RNA polymerase II-associated protein in yeast^36^. The yeast Rtf1 protein is not only essential for Set1-mediated histone H3 methylation but also required for Dot-mediated methylation of histone H3^37^. Moreover, there were also a few mutants with decreased synphilin-1 inclusion formation that affect histone H3 methylation via the COMPASS (Complex Proteins Associated with Set1) complex, such as *swd1Δ*, *swd3Δ*, and *orc2Δ* (Fig. 3a,b). Whether this observation indicates that the Cdc73/Paf1 complex has a direct role in synphilin-1 inclusion formation needs to be further investigated, since the decreased percentage of cells carrying synphilin-1 inclusions may also be due to a general reduction of expression of the marker proteins.

### Inclusion formation defective mutants showing increased synphilin-1 cytotoxicity effect in yeast cells

**Figure 4:**
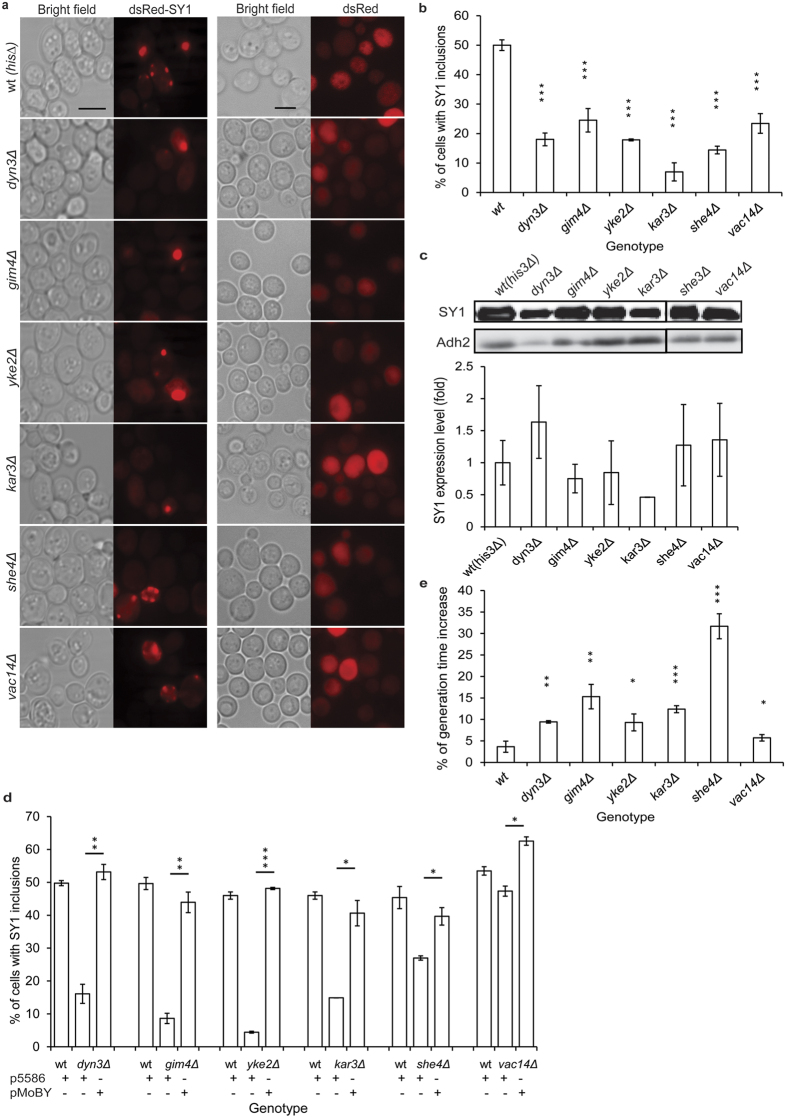
Complementation assays and growth analysis of mutants with decreased inclusion formation capacity. (**a**) Images of selected mutants with a decreased number of cells showing synphilin-1 (SY1) inclusions and the corresponding control mutant strains with only dsRed expression. Left panel, bright field; right panel, dsRed-synphilin-1 or dsRed. Scale bar, 5 μm. (**b**) Quantification of the percentage of cells with synphilin-1 inclusions in WT and mutants. Values are normalized such that the percentage of cells with inclusions in WT cells is set to 50%. A value of 0% means that there are no cells of that mutant with synphilin-1 inclusions. Error bars represent standard deviation from triplicates each containing about 200 cells. (**c**) Western blot analysis of the WT (*his3Δ*) strain and mutants expressing synphilin-1. Immunodetection was performed using primary antibodies directed against synphilin-1 or Adh2, which served as internal control protein. The quantification of synphilin-1 expression levels is shown. Error bars represent standard deviation from triplicates samples. Asterisks denote significant differences between WT and mutants (Student’s *t*-test): *P, 0.05; **P, 0.01; ***P, 0.001. (**d**) Complementation assays further confirmed that the reduced inclusion formation capacity of the mutants is solely due to the lack of function caused by the corresponding deletion. The complementation assays were performed with MoBY plasmids and the empty vector control (p5586). Error bars represent standard deviation from triplicates containing about 200 cells. (**e**) An increased synphilin-1 cytotoxicity effect (prolonged generation time) was observed from the selected mutants. The generation times of mutants expressing dsRed-synphilin-1 and solely dsRed (control) were measured at 30 °C. The differences of generation time increase between WT and mutants are shown. Data are represented as mean ± standard deviation SD. Asterisks denote significant differences between samples (Student’s *t*-test): *P, 0.05; **P, 0.01; ***P, 0.001.

It was previously demonstrated that synphilin-1 inclusions are beneficial and have a positive effect on viability of human cell lines^23^ and humanized yeast cells^7^. To test this further, we selected mutants of different cellular functional groups from the screening and analyzed their growth. For each of these mutants, the synphilin-1 inclusion phenotype was manually checked (Fig. 4a) and this confirmed the decreased capacity to form inclusions (Fig. 4b). In addition, we further measured the expression of synphilin-1 by Western blot, results showed no significant difference in the mutants as compared to the wild type (P > 0.05, Student’s *t*-test, Fig. 4c). Furthermore, we performed complementation assays using Molecular Barcoded Yeast (MoBY) plasmids and this established that the reduced inclusion formation capacity is indeed solely due to the lack of function caused by the corresponding deletion in the mutants (Fig. 4d). Intriguingly, when assessing the growth rate all mutants showed a significantly prolonged generation time (Student’s *t*-test, P ≤ 0.05), which is indicative for a synthetic sick effect when the expression of dsRed-synphilin-1 is combined with the deletion of these genes. No such effect was observed upon expression of solely dsRed, which served as control (Fig. 4e). This result strongly supports the hypothesis that the formation and maturation of inclusions is a cytoprotective process that helps to sequester soluble cytotoxic misconformers into inert deposits^38^.

### Mutants displaying an increased percentage of cells carrying synphilin-1 inclusions

**Figure 5:**
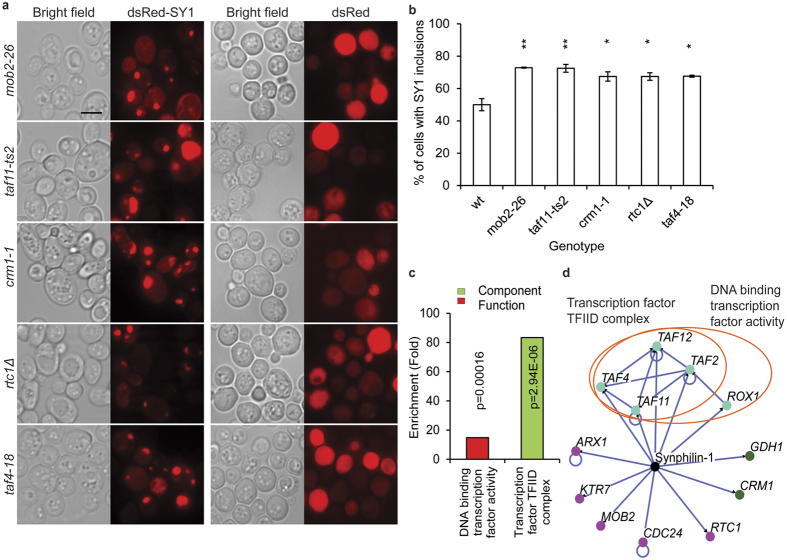
Mutants identified from the HCI screening with an increased percentage of cells carrying synphilin-1 (SY1) inclusions. (**a**) Representative images of strong hits from the imaging based screening showing cells with expression of dsRed-synphilin-1 or dsRed alone. Left panel, bright field; right panel, dsRed-synphilin-1 or dsRed. Scale bar, 5 μm. (**b**) Quantification of percentage of cells with inclusions in WT and mutants. Values are normalized such that the percentage of cells with inclusions in WT cells is set to 50%. Error bars represent standard deviation from triplicates of about 200 cells each. Asterisks denote significant differences between WT and mutants (Student’s *t*-test): *P, 0.05; **P, 0.01; ***P, 0.001. (**c**) Functional enrichment analysis of mutants displaying increased synphilin-1 inclusion formation. Confirmed mutants were analyzed for enrichment of GO functional categories. Enriched groups were scored by comparing to a background list of SGA-V2+ ts-V5 array. Functional groups are marked with different colors. (**d**) Network analysis of mutants with an increased percentage of cells with synphilin-1 inclusions. Mutants showing more cells with synphilin-1 inclusions were grouped into modules based on their known physical interactions and published information of the cellular components. The cellular components are shown in different colored nodes. The circles indicate subunits or protein complexes.

Our screening also identified 15 mutants that showed an increased percentage of cells with dsRed-synphilin-1 inclusions. In each case, the phenotype of the mutant was confirmed by manual analysis. Representative images of strong hits and their quantitative difference from wild-type cells are shown in Fig. 5a,b. Interestingly, when measuring the fluorescence signal intensity many mutants showed elevated levels as compared to the wild type strain, which suggests that the increased inclusion formation phenotype might be caused by an elevated level of synphilin-1 expression (Supplementary Table S6). GO term analysis of these mutants revealed an enrichment for the transcription factor IID (TFIID) complex components (*TAF2*, *TAF4*, *TAF11* and *TAF12*; P = 2.94E-06) (Fig. 5c,d and Supplementary Table S7). Hence, it will be informative to elucidate whether the increased inclusion formation phenotype is caused by a general transcription defect or whether it is the effect of dysregulation of specific downstream targets of the TFIID complex. For six mutants, i.e. *crm1-1, rtc1Δ, cdc24-3, arx1Δ rox1Δ* and *gdh1Δ,* their fluorescence intensities of the dsRed-synphilin-1 fusion were similar to that of the wild type strain. Here, the presence of *GDH1*, a NADP(+)-dependent glutamate dehydrogenase, is especially intriguing. Indeed, changes in the activity and regulation of human GDH have been reported in a number of conditions that lead to neurodegeneration^39^.

In summary, we developed and validated a yeast HCI screening approach for the fluorescently tagged human synphilin-1 protein. This genome-wide imaging screening enabled us to identify cellular components that regulate synphilin-1 aggregation with unexpected functionalities. We found that dynein complex, dynactin, prefoldin complex, the Ctf18 RFC-like complex, the RNA polymerase complex and the Cdc73/Paf1 complex are required for the formation of synphilin-1 inclusions. These results gave an unbiased global view on the complexity of the machinery underlying inclusion formation by synphilin-1 and provided further supports on the hypothesis that the synphilin-1 inclusion formation has a cytoprotective effect to the cell. Synphilin-1 was found to be one of the constituents of Lewy bodies, a hallmark for Parkinson’s disease^40^. Our findings acquired from the yeast model, especially genes/pathways with homologues in human, provided valuable clues concerning the machinery controlling synphilin-1 inclusion formation. Further investigation of these human homologue hits can provide novel insights for our understanding of the mechanisms of pathogenesis causing Parkinson’s disease as well as other protein folding disorders.

## Materials and Methods

### Screening design

#### Yeast strains

The strain Y7092 was used as the SGA starting strain for query strain constructions. The plasmid pYX212-dsRed-synphilin-1 was described previously^7^ and used for expressing N-terminally tagged dsRed-synphilin-1 in the query strain. The yeast single gene knock-out collection (SGA-V2) and the essential gene temperature sensitive allele collection (ts-V5)^41^ are kind gifts from Prof. Charlie Boone. Detailed information regarding the strains used in this study is listed in the Supplementary Table S1. Plasmids p4339, pSG32, pYM28 were used to amplify the natMX4, hphMX4 and EGFP cassettes, respectively. A full list of plasmids used in this study is shown in the Supplementary Table S2.

#### Construction of a genome-wide collection of S. cerevisiae single deletion mutants expressing synphilin-1-dsRed

Query strains were constructed by transferring the pYX212-dsRed-synphilin-1 (sample) into the yeast Y7092 query background. Further, the query strain with synphilin-1-dsRed marker was combined with the SGA-V2 single gene knock-out collection and the ts-V5 array by an automatic synthetic genetic array (SGA) method^8^,^9^,^10^. A control set of the same collection was constructed by introducing the plasmid pYX212-dsRed (control) into both collections. A Singer RoToR HDA robot (Singer Instrument) was used for all the pinning steps for collection handling.

#### Genome-wide high content screening with synphilin-1 inclusions

The collections obtained from the previous steps were stored at −80 °C in 96-well plates. Cells (both single and the ts mutants) were pre-cultured in 200 μl minimal selective SC-Ura liquid medium with antibiotics at 30 °C (22 °C for ts mutants) for 3 days without shaking. Subsequently, each pre-culture was diluted to a starting OD_600nm_ around 0.1 in a 200 μl culture in SC-Ura medium. After 12 h (16 h for ts mutants) growing with shaking at 30 °C (for both single and ts mutants), cells were fixed by adding 20 μl 37% formaldehyde to a final concentration of 3.7% for 30 min at room temperature. Then cells were washed twice with 1xPBS (4,000 rpm for 3 min). Finally, the cells were re-suspended in 200 μl 1xPBS and stored at 4 °C overnight. For imaging, 9 μl re-suspended cells were transferred to new 96-well glass bottom plates (Matri-plate) with 200 μl 1 × PBS in each well. The OD_600nm_ values were adjusted to 0.06–0.08, which corresponds to about 4–8 × 10^4^ cells. The cells in the 96-well plate were spun down at 400 rpm for 45 s, and kept in dark for 30 min before imaging (Texas Red channel, Exposure time: 400 ms) with the automated cellular imaging system ImageXpress MICRO, Molecular Devices Corporation (MDC).

#### Phenotypes

Based on the dsRed-labelled synphilin-1 fluorescence signal, cells expressing synphilin-1 have two distinct phenotypes i.e. a diffused or an aggregated phenotype. The percentage of cells with synphilin-1 inclusion was defined as the amount of cells with synphilin-1 inclusions among all cells showing synphilin-1-dsRed fluorescent signal. The percentage of cells with synphilin-1 inclusions was recorded as the phenotypic readout from the screening. To automatically quantify the synphilin-1 aggregation phenotype, a customized sub-program of the software MetaXpress (MDC) was applied on the obtained images for quantification of percentage of cells carrying synphilin-1 inclusions and to measure the average fluorescence intensity within the cells. Parameters used for the software quantification are set as follows: the signal intensity threshold for cell isolation: ≥300 grayscale units difference between cellular regions and the background of the image; the threshold for synphilin-1 inclusion isolation: ≥400 grayscale units difference between inclusion regions and cellular regions.

#### Verification of mutants and complementation assay

All mutants that showed statistically significant differences from the wild type were re-streaked and tested manually in triplicates to confirm the phenotypic differences observed in the screening. Statistical analysis was conducted using the Student’s *t*- test, and P ≤ 0.05 was used as the cutoff for selecting significant hits. At least 300 cells were counted in the manual confirmation for each mutant. For manual confirmation, cells were grown from starting OD_600nm_ of 0.1 in 15 ml Falcon tubes for 20 h (22 h for TS mutants), subsequently fixed and washed in 1xPBS as described above. All potential hits generated from our high content screening were manually verified by using conventional microscopy. The aggregation phenotype of any high content screening hit, which cannot be repeated in our manual conformation, will be excluded from the final confirmed hits list. To handle the difference between our high content screening and the manual confirmation results, we used the actual values from the manual confirmation as the final results showed in supplementary tables. Complementation assays were performed with a selected group of mutants using the corresponding MoBY plasmids and the empty vector control^42^.

#### Functional enrichment and interaction network analysis

The functional enrichment analysis was based on the results from Gene Ontology Term Finder. P-values were calculated using a hypergeometric distribution with multiple hypothesis correction as described in Boyle *et al.*^43^. Manually confirmed hits (Supplementary Table S3) were analyzed for enrichment of GO biological processes, cellular components and molecular functions by comparison to a background set list of SGA-V2 (4368 genes) plus ts-V5 array (787 ts alleles, covering 497 essential genes) with a P-value cut-off P ≤ 0.05.

The synphilin-1 interaction network diagrams were extracted from the interaction analysis by using Osprey 1.2.0^44^ and the physical interactions between confirmed hits were added according to the BioGRID interaction database^45^. Osprey extracts all experimental interaction data (including large-scale survey and classical genetics) from the BioGRID interaction database, and represents genes as nodes and interactions as edges between nodes. A filter was set for excluding any genetic interactions between the hits, and only presented physical interactions in the diagrams.

### Microscopy

Cell images were obtained by using a Zeiss Axio Observer.Z1 inverted microscope with 100x oil objectives (NA1.4). Filter sets used were GFP, dsRed, Texas Red and DAPI.

### Western blot analysis

Protein extractions and Western blot analysis were performed as previously described^7^. The primary antibodies used were specific for synphilin-1 (Sigma Aldrich, St. Louis, MO, USA) and Adh2 (Merck-Millipore, Overijse, Belgium).

### Synphilin-1 cytotoxicity assay

The synphilin-1 cytotoxicity assays were performed by micro-cultivation experiments in triplicate at 30 °C using the Bioscreen C system (Labsystems Oy, Helsinki, Finland). The optical density was measured every 30 min for 72 h. The significant test was performed and p-values were calculated as described previously^19^.

### Statement on data and reagent availability

Strains are available upon request. File Supplementary Table S1 contains genotypes for each individual strain used. File Supplementary Table S2 contains plasmids used in this study. Full lists of confirmed hits involved in synphilin-1 inclusion formation and their functional enrichment analysis results are listed in File Supplementary Table S3, S4, S6 and S7. File Supplementary Table S5 contains the homologues of yeast genes with function enriched. Mutants showing a decrease in both the percentage of cells with synphilin-1 inclusion and fluorescence intensity are listed in Supplementary Table S8.

## Additional Information

**How to cite this article**: Zhao, L. *et al.* A genome-wide imaging-based screening to identify genes involved in synphilin-1 inclusion formation in *Saccharomyces cerevisiae. Sci. Rep.* **6**, 30134; doi: 10.1038/srep30134 (2016).

## Supplementary Material

Supplementary Information
