# Supplementary material for: A genome-wide imaging-based screening to identify genes involved in synphilin-1 inclusion formation in Saccharomyces cerevisiae
Source: Sci Rep. 2016 Jul 21;6:30134. doi: 10.1038/srep30134 (PMC4954962; doi:10.1038/srep30134)
Supplement: Supplementary Information [file srep30134-s1.pdf]

A genome-wide imaging-based screening to identify genes involved in synphilin-1 inclusion formation in *Saccharomyces cerevisiae*

Lei Zhao[1], Qian Yang[1], Ju Zheng[2], Xuefeng Zhu[3], Xinxin Hao[4], Jia Song[1], Tom Lebacq[2], Vanessa Franssens[2], Joris Winderickx[2]*, Thomas Nystrom[4]*, Beidong Liu[1,4]*

[1] School of Life Science and Technology, Harbin Institute of Technology, Harbin 150001, China

[2] Department of Biology, Functional Biology, KU Leuven, 3001 Heverlee, Belgium

[3] Department of Medical Biochemistry and Cell Biology, University of Gothenburg, S-405 30, Göteborg, Sweden

[4] Department of Chemistry and Molecular Biology, University of Gothenburg, S-413 90, Göteborg, Sweden

*Correspondence: beidong.liu@cmb.gu.se,

thomas.nystrom@cmb.gu.se,

Joris.Winderickx@bio.kuleuven.be

Table of Contents

Supplementary Tables S1: Strains used in this study
Supplementary Tables S2: Plasmids used in this study
Supplementary Tables S3: Mutants with manually confirmed decreased synphilin-1 inclusions phenotype
Supplementary Tables S4: Functional enrichment analysis of confirmed hits with decrease of cells with synphilin-1 inclusion
Supplementary Tables S5: Human homologues of functional enriched yeast genes
Supplementary Tables S6: Mutants with manually confirmed increased synphilin-1 inclusions phenotype
Supplementary Tables S7: Functional enrichment analysis of confirmed hits with increase of cells with synphilin-1 inclusion
Supplementary Tables S8: Mutants showed significant decrease in both the percentage of cells with synphilin-1 inclusion and the fluorescence intensity

Supplementary Figure S1: Manual confirmation status of hits from HCI screening

SUPPLEMENTARY TABLES

Supplementary Table S1: Strains used in this study

Strain Name	Genotype and markers	Background/Source
BY4741 (WT)	*MATa his3Δ1 leu2Δ0 ura3Δ0 met15Δ0*	EUROSCARF; Wt control for strains constructed by PCR approach
Y7092-pSY285	*MATα can1Δ::STE2pr-Sp_his5 lyp1Δ ura3Δ0 leu2Δ0 his3Δ1 met15Δ0 LYS2+* pYX212-dsRed-SY1 (URA+)	This study, query strain
Y7092-pSY244	*MATα can1Δ::STE2pr-Sp_his5 lyp1Δ ura3Δ0 leu2Δ0 his3Δ1 met15Δ0 LYS2+* pYX212-dsRed (URA+)	This study, query strain, Control for strain expressing synphilin-1
BY4741-pSY285	BY4741 with pYX212-dsRed-SY1 (URA+)	This study
BY4741-pSY244	BY4741 with pYX212-dsRed (URA+)	This study, Control for strain expressing synphilin-1
BY4741-pSY285'	BY4741 with pYX212-dsRed-SY1 (Hyg+)	This study
BY4741-pSY244'	BY4741 with pYX212-dsRed (Hyg+)	This study, Control for strain expressing synphilin-1
his3Δ-244	*MATa his3Δ::kanMX4 can1Δ::STE2pr-Sp_his5 lyp1Δ ura3Δ0 leu2Δ0 met15Δ0 LYS2+* pYX212-dsRed (URA+)	This study; Wt control for strains expressing synphilin-1
his3Δ-285	*MATa his3Δ::kanMX4 can1Δ::STE2pr-Sp_his5 lyp1Δ ura3Δ0 leu2Δ0 met15Δ0 LYS2+* pYX212-dsRed-SY1 (URA+)	This study; Wt control for strains constructed by crossing
dyn3Δ-285	*MATa dyn3Δ::kanMX4 can1Δ::STE2pr-Sp_his5 lyp1Δ ura3Δ0 leu2Δ0 met15Δ0 LYS2+* pYX212-dsRed-SY1 (URA+)	This study
gim4Δ-285	*MATa gim4Δ::kanMX4 can1Δ::STE2pr-Sp_his5 lyp1Δ ura3Δ0 leu2Δ0 met15Δ0 LYS2+* pYX212-dsRed-SY1 (URA+)	This study
yke2Δ-285	*MATa yke2Δ::kanMX4 can1Δ::STE2pr-Sp_his5 lyp1Δ ura3Δ0 leu2Δ0 met15Δ0 LYS2+* pYX212-dsRed-SY1 (URA+)	This study
kar3Δ-285	*MATa kar3Δ::kanMX4 can1Δ::STE2pr-Sp_his5 lyp1Δ ura3Δ0 leu2Δ0 met15Δ0 LYS2+*	This study

	pYX212-dsRed-SY1 (URA+)	
she4Δ-285	*MATa she4Δ::kanMX4 can1Δ::STE2pr-Sp_his5 lyp1Δ ura3Δ0 leu2Δ0 met15Δ0 LYS2+*<br>pYX212-dsRed-SY1 (URA+)	This study
vac14Δ-285	*MATa vac14Δ::kanMX4 can1Δ::STE2pr-Sp_his5 lyp1Δ ura3Δ0 leu2Δ0 met15Δ0 LYS2+*<br>pYX212-dsRed-SY1 (URA+)	This study
idh1Δ-285	*MATa idh1Δ::kanMX4 can1Δ::STE2pr-Sp_his5 lyp1Δ ura3Δ0 leu2Δ0 met15Δ0 LYS2+*<br>pYX212-dsRed-SY1 (URA+)	This study
cbc2Δ-285	*MATa cbc2Δ::kanMX4 can1Δ::STE2pr-Sp_his5 lyp1Δ ura3Δ0 leu2Δ0 met15Δ0 LYS2+*<br>pYX212-dsRed-SY1 (URA+)	This study
ctf18Δ-285	*MATa ctf18Δ::kanMX4 can1Δ::STE2pr-Sp_his5 lyp1Δ ura3Δ0 leu2Δ0 met15Δ0 LYS2+*<br>pYX212-dsRed-SY1 (URA+)	This study
srb2Δ-285	*MATa srb2Δ::kanMX4 can1Δ::STE2pr-Sp_his5 lyp1Δ ura3Δ0 leu2Δ0 met15Δ0 LYS2+*<br>pYX212-dsRed-SY1 (URA+)	This study
idh2Δ-285	*MATa idh2Δ::kanMX4 can1Δ::STE2pr-Sp_his5 lyp1Δ ura3Δ0 leu2Δ0 met15Δ0 LYS2+*<br>pYX212-dsRed-SY1 (URA+)	This study
rtg3-285	MATa *rtg3Δ*::kanMX4 can1Δ::STE2pr-Sp_his5 lyp1Δ ura3Δ0 leu2Δ0 met15Δ0 LYS2+<br>pYX212-dsRed-SY1 (URA+)	This study
irc14-285	*MATa irc14Δ::kanMX4 can1Δ::STE2pr-Sp_his5 lyp1Δ ura3Δ0 leu2Δ0 met15Δ0 LYS2+*<br>pYX212-dsRed-SY1 (URA+)	This study
tid3-1-285	*MATa tid3-1::kanMX4 can1Δ::STE2pr-Sp_his5 lyp1Δ ura3Δ0 leu2Δ0 met15Δ0 LYS2+*<br>pYX212-dsRed-SY1 (URA+)	This study

cdc3-1-285	*MATa cdc3-1::kanMX4 can1Δ::STE2pr-Sp_his5 lyp1Δ ura3Δ0 leu2Δ0 met15Δ0 LYS2+* pYX212-dsRed-SY1 (URA+)	This study
his3Δ	BY4741 with *his3Δ::KanMX4*	SGA-V2 collection, Boone lab
dyn3Δ	BY4741 with *dyn3Δ::KanMX4*	SGA-V2 collection, Boone lab
gim4Δ	BY4741 with *gim4Δ::KanMX4*	SGA-V2 collection, Boone lab
yke2Δ	BY4741 with yke2*Δ::KanMX4*	SGA-V2 collection, Boone lab
kar3Δ	BY4741 with *kar3Δ::KanMX4*	SGA-V2 collection, Boone lab
she4Δ	BY4741 with *she4Δ*::*KanMX4*	SGA-V2 collection, Boone lab
vac14Δ	BY4741 with *vac14Δ::KanMX4*	SGA-V2 collection, Boone lab
dyn3Δ-SM	BY4741 with *dyn3Δ::KanMX4* +pYX212-dsRed-SY1 (Hyg+) +MoBY-DYN3(URA+)	This study
gim4Δ-SM	BY4741 with *gim4Δ::KanMX4* +pYX212-dsRed-SY1 (Hyg+) +MoBY-GIM4(URA+)	This study
yke2Δ-SM	BY4741 with *yke2Δ::KanMX4* +pYX212-dsRed-SY1 (Hyg+) +MoBY-YKE2(URA+)	This study
kar3Δ-SM	BY4741 with *kar3Δ::KanMX4* +pYX212-dsRed-SY1 (Hyg+) +MoBY-KAR3(URA+)	This study
she4Δ-SM	BY4741 with *she4Δ::KanMX4* +pYX212-dsRed-SY1 (Hyg+) +MoBY-SHE4(URA+)	This study
vac14Δ-SM	BY4741 with *vac14Δ::KanMX4* +pYX212-dsRed-SY1 (Hyg+) +MoBY-VAC14(URA+)	This study
his3Δ-SP	BY4741 with *his3Δ::KanMX4* +pYX212-dsRed-SY1 (Hyg+)+p5586(URA+)	This study
dyn3Δ-SP	BY4741 with *dyn3Δ::KanMX4* +pYX212-dsRed-SY1 (Hyg+)+p5586(URA+)	This study
gim4Δ-SP	BY4741 with *gim4Δ::KanMX4* +pYX212-dsRed-SY1 (Hyg+)+p5586(URA+)	This study
yke2Δ-SP	BY4741 with *yke2Δ::KanMX4* +pYX212-dsRed-SY1 (Hyg+)+p5586(URA+)	This study
kar3Δ-SP	BY4741 with *kar3Δ::KanMX4* +pYX212-dsRed-SY1 (Hyg+)+p5586(URA+)	This study

she4Δ-SP	BY4741 with *she4Δ::KanMX4* +pYX212-dsRed-SY1 (Hyg+)+p5586(URA+)	This study
vac14Δ-SP	BY4741 with *vac14Δ::KanMX4* +pYX212-dsRed-SY1 (Hyg+)+p5586(URA+)	This study
SGA-V2 collection	BY4741 with *xxxΔ::KanMX4* collection	Boone lab
Ts-V5 collection	BY4741 background, essential gene temperature sensitive allele collection	Boone lab
S2YDSY1 collection	SGA-V2 with pYX212-dsRed-synphilin-1	This study
S2YD collection	SGA-V2 with pYX212-dsRed (control for S2YDSY1 collection	This study
Sts5YDSY1 collection	Ts-V5 with pYX212-dsRed-synphilin-1	This study
Sts5YD collection	Ts-V5 with pYX212-dsRed (control for Sts5YDSY1 collection)	This study

Supplementary Table S2: Plasmids used in this study

Plasmid Name	Genotype and markers	Background/Source
pYX285	pYX212-SY1-dsRed, (URA3+, AmpR)	BÜTTNER et al. 2010
pYX244	pYX212-dsRed, (URA3+, AmpR)	BÜTTNER et al. 2010
pYX285'	pYX212-SY1-dsRed, (Hygromycin B+, AmpR)	This study
pYX244'	pYX212-dsRed, (Hygromycin B+, AmpR)	This study
p5586	p5472+URA3+CEN+MAGIC (URA3+, tetracycline+, kanamycin+, chloramphenicol+)	MoBY-ORF library
pMoBY-DYN3	p5586-*DYN3* ORF (URA3+, Hygromycin B+, tetracycline+, kanamycin+, chloramphenicol+)	MoBY-ORF library
pMoBY-GIM4	p5586-*GIM4* ORF (URA3+, Hygromycin B+, tetracycline+, kanamycin+, chloramphenicol+)	MoBY-ORF library
pMoBY-YKE2	p5586-*YKE2* ORF (URA3+, Hygromycin B+, tetracycline+, kanamycin+, chloramphenicol+)	MoBY-ORF library
pMoBY-KAR3	p5586-*KAR3* ORF(URA3+, Hygromycin B+, tetracycline+, kanamycin+, chloramphenicol+)	MoBY-ORF library

pMoBY-SHE4	p5586-*SHE4* ORF (URA3+, Hygromycin B+, tetracycline+, kanamycin+, chloramphenicol+)	MoBY-ORF library
pMoBY-VAC14	p5586-*VAC14* ORF (URA3+, tetracycline+, kanamycin+, chloramphenicol+)	MoBY-ORF library
pSG32	(Hygromycin B+)	For hphMX4 amplify

Supplementary Table S3: Mutants with manually confirmed decreased synphilin-1 inclusions phenotype

Number	Mutant	Normalized percentage of cells with SY1 inclusions (%)	Relative intensity	Intensity *t*-test (P value)
0	wt	50.00	100.00	
1	*cbc2Δ*	0.20	22.71	0.0702
2	*ctf18Δ*	1.37	61.44	0.2371
3	*tid3-1*	1.70	67.78	0.1161
4	*idh2Δ*	1.70	74.41	0.3265
5	*cdc3-1*	2.66	68.16	0.0531
6	*srb2Δ*	2.76	103.01	0.5860
7	*rtg3Δ*	2.91	42.39	0.0855
8	*irc14Δ*	3.24	62.78	0.0980
9	*idh1Δ*	3.41	68.76	0.0832
10	*cdc5-1*	4.22	70.19	0.0616
11	*cdc73Δ*	4.23	70.79	0.1479
12	*bre1Δ*	4.41	70.47	0.1634
13	*rad14Δ*	4.83	123.66	0.0013
14	*bap2Δ*	5.84	61.73	0.2459
15	*psf1-1*	5.90	98.45	0.6070
16	*abd1-5*	6.11	59.33	0.0731
17	*gpi19-2*	6.18	82.48	0.2261
18	*act1-112*	6.23	62.15	0.1426
19	*cdc20-1*	6.38	58.30	0.1508
20	*jnm1Δ*	6.67	71.07	0.0656
21	*rfc2-1*	6.77	89.79	0.3747
22	*kar3Δ*	6.99	47.55	0.1204
23	*soh1Δ*	7.05	99.93	0.9894
24	*pac10Δ*	7.27	88.78	0.6408
25	*rpn12-1*	7.29	93.00	0.1913
26	*spt14-1-10c*	7.35	92.17	0.2130
27	*orc2-2*	7.45	97.09	0.6545
28	*cdc53-1*	7.67	60.00	0.0983

29	*act1-133*	7.87	62.94	0.0613
30	*rpn10Δ*	8.12	82.93	0.3363
31	*hur1Δ*	8.28	96.68	0.8663
32	*pmr1Δ*	8.38	80.18	0.2948
33	*ctf4Δ*	8.51	47.63	0.1020
34	*act1-105*	8.73	64.67	0.1957
35	*pol1-13*	8.93	88.12	0.3093
36	*spe3Δ*	8.99	86.02	0.1787
37	*smc1-2*	9.23	77.97	0.0543
38	*spt8Δ*	9.30	67.17	0.2603
39	*act1-3*	9.65	86.73	0.0884
40	*mcm3-1*	10.07	74.84	0.2017
41	*mms22Δ*	10.15	60.34	0.3571
42	*ira2Δ*	10.45	63.52	0.1043
43	*apc11-22*	10.47	72.38	0.1282
44	*gpr1Δ*	10.48	92.81	0.5443
45	*pds5-1*	10.92	58.63	0.1094
46	*isc1Δ*	10.99	93.85	0.9580
47	*rvs161Δ*	11.27	102.33	0.7764
48	*stu1-6*	11.38	90.15	0.2789
49	*est1Δ*	11.60	83.33	0.4955
50	*rfa1-m2*	11.92	90.36	0.1263
51	*rpo21-1*	11.99	62.24	0.0547
52	*stu2-10*	12.64	108.87	0.7958
53	*mms1Δ*	12.78	65.37	0.3096
54	*rcy1Δ*	13.11	64.18	0.1897
55	*act1-125*	13.28	66.49	0.1024
56	*ctf8Δ*	13.85	61.49	0.0987
57	*she4Δ*	14.42	65.36	0.2147
58	*asf1Δ*	14.42	78.89	0.2732
59	*cdc20-3*	14.42	60.24	0.0866
60	*act1-129*	14.55	94.62	0.1015
61	*pib2Δ*	14.59	108.39	0.7504
62	*aro2Δ*	14.67	81.65	0.3638
63	*lat1Δ*	14.96	73.78	0.0602
64	*leo1Δ*	15.18	98.54	0.1710
65	*arg2Δ*	15.37	92.61	0.3546
66	*rad52Δ*	15.59	73.62	0.2094
67	*csg2Δ*	15.81	92.07	0.7368
68	*lge1Δ*	15.85	76.55	0.4217
69	*aah1Δ*	16.17	76.85	0.3225
70	*rpe1Δ*	16.17	91.34	0.9704
71	*rad27Δ*	16.20	91.37	0.0791
72	*rtf1Δ*	16.37	75.43	0.0947

73	*cdc15-2*	16.61	86.59	0.1059
74	*clb5Δ*	16.71	81.56	0.9800
75	*pcf11-ts2*	16.81	75.80	0.1105
76	*pde2Δ*	16.91	91.56	0.3362
77	*nip100Δ*	17.01	67.94	0.5268
78	*dia2Δ*	17.34	150.63	0.0421
79	*sec66Δ*	17.44	89.21	0.1357
80	*yta7Δ*	17.54	97.63	0.6665
81	*sub1Δ*	17.64	79.22	0.6241
82	*yke2Δ*	17.85	89.72	0.6966
83	*gwt1-20*	17.97	107.70	0.9268
84	*dyn3Δ*	18.03	81.67	0.3326
85	*arv1Δ*	18.52	95.78	0.8862
86	*sla1Δ*	19.45	73.56	0.5605
87	*spf1Δ*	19.51	82.87	0.0528
88	*swd1Δ*	19.52	66.96	0.7769
89	*bts1Δ*	19.59	80.62	0.8082
90	*ubp15Δ*	19.85	64.47	0.0589
91	*dyn1Δ*	19.98	79.14	0.5109
92	*kem1Δ*	20.12	59.61	0.1279
93	*num1Δ*	20.14	81.76	0.0780
94	*swi6Δ*	20.27	62.52	0.0688
95	*chl1Δ*	20.96	78.81	0.4542
96	*gtr2Δ*	21.10	89.08	0.2366
97	*dep1Δ*	21.28	75.51	0.3573
98	*hex3Δ*	22.09	92.89	0.7206
99	*rtt109Δ*	22.17	103.60	0.1670
100	*ssk1Δ*	22.52	64.47	0.2329
101	*lrp1Δ*	22.63	107.11	0.2375
102	*ldb18Δ*	22.73	75.88	0.3947
103	*ade1Δ*	22.86	73.75	0.5854
104	*vac14Δ*	23.42	96.55	0.6339
105	*pac1Δ*	23.55	76.11	0.1951
106	*pdb1Δ*	23.98	60.15	0.1658
107	*ost4Δ*	24.17	93.49	0.3114
108	*swd3Δ*	24.30	79.39	0.7011
109	*spe2Δ*	24.32	80.62	0.5859
110	*gim4Δ*	24.53	88.24	0.2892
111	*thr4Δ*	24.54	73.25	0.1908
112	*las21Δ*	24.72	93.23	0.3713
113	*sac1Δ*	26.76	79.32	0.2403
114	*ded1-199*	27.03	67.87	0.0536
115	*stt3-7*	27.23	102.65	0.8626
116	*tps1Δ*	27.25	57.46	0.1786

117	*ecm8Δ*	27.64	81.81	0.8692
118	*hoc1Δ*	27.84	77.74	0.4873
119	*pac11Δ*	28.55	87.05	0.1662
120	*mct1Δ*	28.70	74.61	0.1654
121	*nkp2Δ*	29.14	78.15	0.7418
122	*ecm33Δ*	30.06	75.96	0.9666
123	*nup133Δ*	30.21	88.16	0.7494
124	*gyp1Δ*	30.47	74.13	0.1783
125	*sic1Δ*	30.48	88.67	0.8745
126	*etr1Δ*	31.07	62.67	0.2541
127	*ald6Δ*	32.28	83.63	0.9568
128	*clb2Δ*	32.67	65.62	0.3195
129	*hom6Δ*	33.72	86.16	0.1411
130	*slm4Δ*	34.81	73.46	0.5164
131	*lip5Δ*	34.87	65.44	0.1015
132	*tip1Δ*	35.50	73.06	0.5078
133	*syg1Δ*	36.67	59.63	0.0813

* $P \leq 0.05$ indicates significant signal intensity difference between wild-type and mutant

Supplementary Table S4: Functional enrichment analysis of confirmed hits with decrease of cells with synphilin-1 inclusion

Terms	Gene(s) annotated to the term
Microtubule cytoskeleton organization	CDC15/STU1/NUM1/PAC11/ACT1/NDC80/DYN1/LDB18/STU2/CDC5/JNM1/NIP100/CLB2/CLB5/KAR3
DNA replication	DEP1/RFA1/ORC2/BRE1/PSF1/MCM3/HUR1/RFC2/RAD27/MMS22/CTF18/POL1/DIA2/LGE1/CLB5/CTF4/MMS1
Positive regulation of DNA-templated transcription, elongation	XRN1/RTF1/ASF1/YKE2/CDC73/SUB1/LEO1
Histone modification	DEP1/SWD1/ORC2/SWD3/BRE1/ACT1/RTF1/ASF1/RTT109/SPT8/CDC73/LEO1/LGE1
Sister chromatid segregation	SMC1/CDC20/SOH1/CTF8/RFC2/DYN1/MMS22/PDS5/CTF18/CHL1/CLB2/CTF4/KAR3
Glycolipid biosynthetic process	CSG2/GPI19/LAS21/GWT1/ARV1/SPT14
DNA repair	RFA1/BRE1/RPO21/PSF1/MCM3/SMC1/ACT1/SOH1/RTF1/RFC2/RAD27/NUP133/RTT109/MMS22/CDC73/RAD52/SUB1/PDS5/CTF18/RAD14/POL1/LEO1/CHL1/CTF4/MMS1

Supplementary Table S5: Human homologues of functional enriched yeast genes

Yeast Gene Name	Homologue Standard Name	Homologue Organism	Cross References Identifier	Homologue Diseases	Source
ACT1	ACTA1	H. sapiens	255310	MYOPATHY, CONGENITAL, WITH FIBER-TYPE DISPROPORTION; CFTD	Ensembl
ACT1	ACTA2	H. sapiens	614042	MOYAMOYA DISEASE 5; MYMY5	Ensembl
ACT1	ACTB	H. sapiens	243310	BARAITSER-WINTER SYNDROME 1; BRWS1	Ensembl
ACT1	ACTBL2	H. sapiens			Ensembl
ACT1	ACTC1	H. sapiens	613424	CARDIOMYOPATHY, DILATED, 1R; CMD1R LEFT VENTRICULAR NONCOMPACTION 4, INCLUDED; LVNC4, INCLUDED	Ensembl
ACT1	ACTG2	H. sapiens	155310	VISCERAL MYOPATHY; VSCM	Ensembl
ACT1	ACTR1A	H. sapiens			Ensembl
ACT1	ACTR1B	H. sapiens			Ensembl
ACT1	ACTRT1	H. sapiens			Ensembl
ACT1	ACTRT2	H. sapiens			Ensembl
ACT1	ACTRT3	H. sapiens			Ensembl
ASF1	ASF1A	H. sapiens			Ensembl
ASF1	ASF1B	H. sapiens			Ensembl
BRE1	RNF20	H. sapiens			Ensembl
BRE1	RNF40	H. sapiens			Ensembl
CDC15	MAP3K14	H. sapiens			Ensembl
CDC15	MAP3K15	H. sapiens			Ensembl
CDC15	MAP3K5	H. sapiens			Ensembl
CDC15	MAP3K6	H. sapiens			Ensembl
CDC15	MAP3K8	H. sapiens	211980	LUNG CANCER ALVEOLAR CELL CARCINOMA, INCLUDED	Ensembl
CDC5	PLK1	H. sapiens			Ensembl
CDC5	PLK2	H. sapiens			Ensembl
CDC5	PLK3	H. sapiens			Ensembl
CDC5	PLK4	H. sapiens	616171	MICROCEPHALY AND CHORIORETINOPATHY, AUTOSOMAL RECESSIVE, 2; MCCRP2	Ensembl
CDC73	CDC73	H. sapiens	608266	PARATHYROID CARCINOMA	Ensembl
CDC73	CDC73	H. sapiens	145001	HYPERPARATHYROIDISM 2; HRPT2	Ensembl

CDC73	CDC73	H. sapiens	145000	HYPERPARATHYROIDISM 1; HRPT1	Ensembl
CHL1	BRIP1	H. sapiens	609054	FANCONI ANEMIA, COMPLEMENTATION GROUP J; FANCJ	Ensembl
CHL1	BRIP1	H. sapiens	114480	BREAST CANCER	Ensembl
CHL1	DDX11	H. sapiens	613398	WARSAW BREAKAGE SYNDROME; WABS	Ensembl
CHL1	RTEL1	H. sapiens	616373	PULMONARY FIBROSIS AND/OR BONE MARROW FAILURE, TELOMERE-RELATED, 3; PFBMFT3	Ensembl
CHL1	RTEL1	H. sapiens	615190	DYSKERATOSIS CONGENITA, AUTOSOMAL RECESSIVE 5; DKCB5 DYSKERATOSIS CONGENITA, AUTOSOMAL DOMINANT 4, INCLUDED; DKCA4, INCLUDED	Ensembl
CLB2	CCNA1	H. sapiens			Ensembl
CLB2	CCNA2	H. sapiens			Ensembl
CLB2	CCNB1	H. sapiens			Ensembl
CLB2	CCNB2	H. sapiens			Ensembl
CLB2	CCNB3	H. sapiens			Ensembl
CLB2	CCND1	H. sapiens	193300	VON HIPPEL-LINDAU SYNDROME; VHL VON HIPPEL-LINDAU SYNDROME, MODIFIERS OF, INCLUDED	Ensembl
CLB2	CCND1	H. sapiens	114500	COLORECTAL CANCER; CRC	Ensembl
CLB2	CCND1	H. sapiens	254500	MYELOMA, MULTIPLE AMYLOIDOSIS, SYSTEMIC, INCLUDED; AL, INCLUDED	Ensembl
CLB2	CCND2	H. sapiens	615938	MEGALENCEPHALY-POLYMICROGYRIA-POLYDACTYLY-HYDROCEPHALUS SYNDROME 3; MPPH3	Ensembl
CLB2	CCND3	H. sapiens			Ensembl
CLB2	CCNE1	H. sapiens			Ensembl
CLB2	CCNE2	H. sapiens			Ensembl
CLB2	CCNO	H. sapiens	615872	CILIARY DYSKINESIA, PRIMARY, 29; CILD29	Ensembl
CLB5	CCNA1	H. sapiens			Ensembl
CLB5	CCNA2	H. sapiens			Ensembl

CLB5	CCNB1	H. sapiens			Ensembl
CLB5	CCNB2	H. sapiens			Ensembl
CLB5	CCNB3	H. sapiens			Ensembl
CLB5	CCND1	H. sapiens	193300	VON HIPPEL-LINDAU SYNDROME; VHL VON HIPPEL-LINDAU SYNDROME, MODIFIERS OF, INCLUDED	Ensembl
CLB5	CCND1	H. sapiens	114500	COLORECTAL CANCER; CRC	Ensembl
CLB5	CCND1	H. sapiens	254500	MYELOMA, MULTIPLE AMYLOIDOSIS, SYSTEMIC, INCLUDED; AL, INCLUDED	Ensembl
CLB5	CCND2	H. sapiens	615938	MEGALENCEPHALY-POLYMICROGYRIA-POLYDACTYLY-HYDROCEPHALUS SYNDROME 3; MPPH3	Ensembl
CLB5	CCND3	H. sapiens			Ensembl
CLB5	CCNE1	H. sapiens			Ensembl
CLB5	CCNE2	H. sapiens			Ensembl
CLB5	CCNO	H. sapiens	615872	CILIARY DYSKINESIA, PRIMARY, 29; CILD29	Ensembl
CTF18	CHTF18	H. sapiens			Ensembl
CTF4	WDHD1	H. sapiens			Ensembl
GIM4	PFDN2	H. sapiens			Ensembl
GWT1	PIGW	H. sapiens	616025	HYPERPHOSPHATASIA WITH MENTAL RETARDATION SYNDROME 5; HPMRS5	Ensembl
KAR3	KIF25	H. sapiens			Ensembl
KAR3	KIFC1	H. sapiens			Ensembl
KAR3	KIFC3	H. sapiens			Ensembl
LEO1	LEO1	H. sapiens			Ensembl
MCM3	MCM3	H. sapiens			Ensembl
NDC80	NDC80	H. sapiens			Ensembl
NIP100	CLIP1	H. sapiens			Ensembl
NIP100	CLIP2	H. sapiens			Ensembl
NIP100	CLIP3	H. sapiens			Ensembl
PAC10	VBP1	H. sapiens			Ensembl
PDS5	PDS5A	H. sapiens			Ensembl
PDS5	PDS5B	H. sapiens			Ensembl
POL1	POLA1	H. sapiens	310465	N SYNDROME; NSX	Ensembl
PSF1	GINS1	H. sapiens			Ensembl
RFA1	RPA1	H. sapiens			Ensembl
RFC2	RFC4	H. sapiens			Ensembl
RTF1	RTF1	H. sapiens			Ensembl
SMC1	SMC1A	H. sapiens	300590	CORNELIA DE LANGE	Ensembl

				SYNDROME 2; CDLS2	
SMC1	SMC1B	H. sapiens			Ensembl
SPT14	PIGA	H. sapiens	300868	MULTIPLE CONGENITAL ANOMALIES-HYPOTONIA-SEIZURES SYNDROME 2; MCAHS2	Ensembl
SPT14	PIGA	H. sapiens	300818	PAROXYSMAL NOCTURNAL HEMOGLOBINURIA 1; PNH1	Ensembl
STU2	CKAP5	H. sapiens			Ensembl
SUB1	SUB1	H. sapiens			Ensembl
SWD1	RBBP5	H. sapiens			Ensembl
XRN1	XRN1	H. sapiens			Ensembl
YKE2	PFDN6	H. sapiens			Ensembl

Supplementary Table S6: Mutants with manually confirmed increased synphilin-1 inclusions phenotype

Number	Mutant	Normalized percentage of cells with SY1 inclusions (%)	Relative intensity	Intensity *t*-test (P value)
0	wt	50.00	100.00	
1	*mob2-26*	73.01	141.41	0.0453
2	*taf11-ts2*	72.52	214.15	0.0165
3	*crm1-1*	68.70	110.51	0.5755
4	*rtc1Δ*	68.22	116.51	0.2105
5	*taf4-18*	67.69	141.59	0.0179
6	*cdc24-3*	67.21	113.76	0.6103
7	*taf12-W486*	65.61	163.97	0.0214
8	*arx1Δ*	63.23	124.13	0.5328
9	*ktr7Δ*	62.89	128.45	0.0253
10	*rox1Δ*	61.78	92.71	0.9362
11	*taf12-L464A*	58.07	266.95	0.0045
12	*taf12-9*	57.77	154.13	0.0385
13	*taf12-L446A*	57.71	193.79	0.0061
14	*taf2-1*	57.28	216.78	0.0040
15	*gdh1Δ*	54.82	102.01	0.0978

* P≤0.05 indicates significant signal intensity difference between wild-type and mutant

Supplementary Table S7: Functional enrichment analysis of confirmed hits with increase of cells with synphilin-1 inclusion

Terms	Gene(s) annotated to the term
DNA binding transcription factor activity	TAF2/TAF12/TAF11/TAF4/ROX1
Transcription factor TFIID complex	TAF2/TAF12/TAF11/TAF4

Supplementary Table 8: Mutants showed significant decrease in both the percentage of cells with synphilin-1 inclusion and the fluorescence intensity

Number	Mutant	Normalized percentage of cells with SY1 inclusions (%)	Relative intensity	Intensity *t*-test (P value)
0	wt	50.00	100.00	
1	*sem1Δ*	0.00	61.94	0.0002
2	*dbp5-2*	0.00	34.32	0.0030
3	*cdc10-5*	0.00	42.79	0.0162
4	*cdc40-ts*	0.29	47.33	0.0114
5	*bbp1-2*	0.42	23.54	0.0097
6	*dbp5-1*	0.46	25.95	0.0027
7	*hsl1Δ*	0.61	65.14	0.0442
8	*yhc1-7*	0.98	61.08	0.0135
9	*scd5-D338*	1.37	59.15	0.0303
10	*cks1-38*	1.52	39.71	0.0124
11	*nop2-10*	1.75	51.24	0.0481
12	*spt3Δ*	1.80	70.61	0.0299
13	*cdc28-td*	1.92	38.01	0.0259
14	*spc29-20*	2.04	59.88	0.0106
15	*nip7-1*	2.15	53.83	0.0098
16	*srm1-G282S*	2.21	60.57	0.0328
17	*cdc123-4*	2.28	35.70	0.0030
18	*pri2-1*	2.59	76.96	0.0201
19	*prp18-1202*	3.01	74.75	0.0490
20	*rtg2Δ*	3.09	53.06	0.0280
21	*med6-ts*	3.13	41.44	0.0092
22	*orc3-70*	3.14	67.46	0.0096
23	*cks1-35*	3.41	25.26	0.0200
24	*scd5-PP1D2*	3.47	42.85	0.0133
25	*mtr3-ts*	3.53	51.54	0.0110
26	*stu1-8*	3.56	50.81	0.0171
27	*apc11-13*	3.91	43.41	0.0072
28	*pol1-2*	4.06	67.81	0.0174

29	*orc2-1*	4.06	48.64	0.0205
30	*ded1-95*	4.20	52.68	0.0131
31	*las17-13*	4.83	49.88	0.0460
32	*act1-155*	4.95	52.21	0.0114
33	*act1-132*	5.31	61.03	0.0074
34	*dcc1Δ*	5.73	65.61	0.0094
35	*sup35-td*	6.02	45.82	0.0076
36	*bem2Δ*	6.05	50.21	0.0149
37	*spt6-14*	6.07	49.81	0.0281
38	*slu7-ts2*	6.09	65.87	0.0225
39	*cdc37-ts*	6.19	45.76	0.0036
40	*rmp1-ts*	6.75	54.20	0.0337
41	*cdc48-3*	6.97	58.69	0.0225
42	*rtg1Δ*	7.69	40.06	0.0359
43	*rna15-58*	7.77	71.03	0.0184
44	*cft2-1*	8.01	70.40	0.0286
45	*stu2-11*	8.26	73.30	0.0408
46	*lcb1-10*	8.35	58.60	0.0149
47	*lst8-6*	8.85	68.69	0.0329
48	*elm1Δ*	8.98	46.75	0.0176
49	*stu2-12*	8.99	55.24	0.0116
50	*cik1Δ*	9.02	57.19	0.0047
51	*mnn10Δ*	9.38	46.61	0.0081
52	*mps3-7*	9.40	43.24	0.0333
53	*arp1Δ*	9.44	81.88	0.0034
54	*stp1Δ*	9.83	80.72	0.0305
55	*orc2-4*	9.93	74.93	0.0468
56	*mud2Δ*	10.14	48.14	0.0037
57	*sub2-1*	10.45	35.45	0.0088
58	*ssd1Δ*	10.86	90.76	0.0497
59	*cdc28-4*	10.99	62.28	0.0200
60	*afg2-18*	11.16	57.09	0.0118
61	*spt5-194*	11.17	57.34	0.0382
62	*swi4Δ*	11.31	92.23	0.0495
63	*nup192-15*	11.32	65.10	0.0331
64	*cdc8-2*	12.30	88.38	0.0366
65	*cdc28-13*	12.30	56.84	0.0461
66	*xrs2Δ*	12.32	86.78	0.0159
67	*med8-51*	12.73	59.44	0.0191
68	*pfy1-4*	12.79	46.65	0.0294
69	*mre11Δ*	14.13	66.29	0.0471
70	*bim1Δ*	14.27	49.17	0.0270
71	*cdc33-E72G*	14.33	57.95	0.0094
72	*dam1-19*	14.59	46.89	0.0021

73	*pri1-M4*	15.66	79.81	0.0214
74	*sse1Δ*	15.96	53.16	0.0385
75	*gas1Δ*	17.47	77.04	0.0209
76	*mks1Δ*	20.12	46.09	0.0164
77	*cdc4-3*	20.20	67.39	0.0428
78	*rpo41Δ*	20.34	72.04	0.0171
79	*tfa2-45*	22.67	70.31	0.0223
80	*pol12-ts*	24.92	62.32	0.0186
81	*gcr2Δ*	26.82	41.63	0.0299
82	*enp1-1*	30.34	67.76	0.0337
83	*ume6Δ*	32.50	66.11	0.0210
84	*wbp1-1*	33.11	72.31	0.0313
85	*rio2-1*	35.75	65.69	0.0268
86	*pda1Δ*	36.10	79.11	0.0205

* $P \leq 0.05$ indicates significant signal intensity difference between wild-type and mutant

SUPPLEMENTARY FIGURE

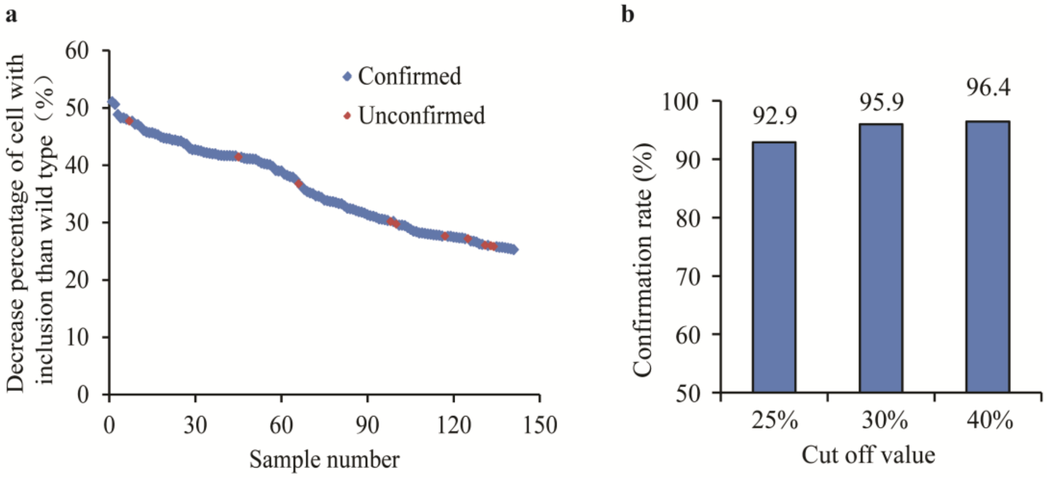

Supplementary Figure S1. Manual confirmation status of hits from HCI screening. a) The distribution of the confirmation status of decreased synphilin-1 inclusion formation hits, sorted based on their difference (from high to low) from the WT strain. Blue dots represented confirmed hits, and red ones represented unconfirmed mutants. b) Confirmation rate of hits with decrease synphilin-1 inclusion formation rate. Bars showed the confirmation rate by manual confirmation with different cut off values.
